# Improving outcomes in single chamber leadless pacemakers: strategies for minimizing vascular complications

**DOI:** 10.1186/s12872-023-03634-9

**Published:** 2023-12-08

**Authors:** Koushan Kouranloo, Joanne Lawson, Angelic Goode, Grahame Goode, Khalid Abozguia

**Affiliations:** 1 University of Liverpool School of Medicine, Ashton St, Liverpool, UK; 2 Royal Liverpool University Hospitals Mount Vernon St, Liverpool, UK; 3 Blackpool Victoria Hospital, NHS Foundation Trust, Blackpool, UK; 4 Marshall Cardiology, Marshall University Joan C. Edwards School of Medicine, Huntington, WV USA

**Keywords:** Ultrasound guided venipuncture, Z suture, Leadless pacemaker, Micra, Vascular complication

## Abstract

**Background:**

Leadless pacemaker therapy is associated with a significant reduction in lead-related complication rate compared to conventional transvenous single chamber pacemaker therapy. However, a significant complication rate of 1.2% was observed in vascular access due to the use of large delivery femoral sheath (27Fr). The aim of this study was to evaluate the effectiveness of real-time ultrasound guidance and Z suture technique in reducing total and major vascular complications in leadless pacemaker therapy.

**Method:**

In this study, we performed a retrospective and prospective analysis of all adverse events associated with leadless pacemaker (Micra) implantation by two operators at a single tertiary center from December 2016 to December 2018. To mitigate the risk of vascular complications, all patients underwent real-time ultrasound-guided venipuncture for vascular access, as well as the application of a Z-suture technique for hemostasis at the end of the procedure. Data were collected on implant indications, implant procedure details, complications, and follow-up information.

**Results:**

In this study, 45 patients with an age range of 24 to 94 years (mean 76 ± 14 years) were recruited, with 21 (46.6%) being female. The pacing indications for the patients included atrial fibrillation (24, 53.3%), vascular (7, 15.5%), infection (9, 20%), cognitive/frailty (3, 6.6%), and occupational (2, 4.4%). The implant procedures were performed under general anesthesia in 6 (13.3%) of the cases, and under local anesthesia and sedation in 39 (86.6%) of the cases. A single deployment was achieved in 43 (95.5%) of the patients, while 2 deployments were required in 2 (4.4%) of the patients. Notably, no vascular or major complications were reported in our cohort of patients.

**Conclusions:**

The results of this observational study indicate that incorporating real-time ultrasound guidance during venipuncture and the use of a Z-suture technique significantly reduce the occurrence of both total and major vascular complications associated with the implantation of leadless pacemaker. However, more robust and larger studies are required in order to confirm these results and implications for clinical practice.

## Introduction

Despite significant advancements in cardiac pacing and resynchronization therapy, a variety of potential acute and chronic complications remain associated with this technology. Transvenous leads and subcutaneous generator pockets are a significant contributor to these complications. Acute complications, such as pneumothorax, lead dislodgement, and pocket hematoma, as well as chronic complications such as lead fractures, skin erosion, and pocket infection leading to septicemia have been reported in the literature. Additionally, the presence of transvenous pacing leads has been associated with an increased risk of deep vein thrombosis, venous obstruction, and tricuspid valve insufficiency [1–5].

The literature suggests a high incidence of post-operative complications related to conventional pacemaker implantation, with estimates as high as 10% [4, 5]. These complications often necessitate re-intervention and may be potentially life-threatening in cases of septicemia and endocarditis, with reported mortality rates ranging between 12% and 31% [1, 3–5].

Leadless pacing was first introduced in the 1970s with the goal of eliminating complications associated with traditional pacemaker pocket and lead systems [6]. Currently, two leadless pacing devices are available clinically, the Nanostim Leadless Pacemaker System (LPS; St. Jude Medical) [3] and the Micra Transcatheter Pacing System (TPS; Medtronic) [3], however, the use of Nanostim LPS is currently halted globally due to issues with battery longevity and risk of cardiac perforation [6].

The Micra Transcatheter Pacing System (TPS) developed by Medtronic is a single-chamber ventricular device measuring at 0.8ml. The device is anchored in the right ventricle using Nitinol fixation tines that are separated from the main electrode. This design results in less fibrosis and is associated with lower pacing thresholds and improved battery longevity [4].

The Micra Transcatheter Pacing System’s introducer is a 27-French outer diameter sheath with a hydrophilic coating. Clinical studies have reported an implantation success rate greater than 99% and a reduction of greater than 60% in major complications compared to traditional pacemakers [1–3].

Vascular complications associated with Micra implantation have been reported at a rate of 1.2% [9]. Considering the size of the introducer and the anatomical variations of the femoral venous and arterial circulation [7], we sought to investigate the importance of ultrasound-facilitated venous access to prevent accidental femoral arterial puncture. In this study, we evaluated the use of the Z suture technique to achieve hemostasis and minimize groin and vascular complications.

## Method

A retrospective and prospective analysis of all patients implanted with the Micra Transcatheter Leadless Pacing System (Medtronic, Minneapolis, USA) at a single tertiary center between December 2016 and December 2018 was conducted. Patient demographics, baseline characteristics, clinical indications, and outcomes were collected and analyzed. Descriptive statistics, including means and standard deviations, were used to present the data. Ethical approval was not required for this study.

The definition of major complications adhered to published literature, which defined it as an event leading to death, hospitalization, prolonged inpatient stay or loss of device function. Vascular complications were specifically defined as including: groin hematoma, retroperitoneal bleeding, pseudoanyuresm, arteriovenous fistula, thrombosis, embolism and the need for blood transfusion, vascular intervention, or vascular surgical intervention as a result of access site complications.

Ultrasound guidance was employed for venous access in all patients. Hemostasis was achieved through the use of Z suture. The procedure of leadless pacing has been previously described in the literature [8]. To reduce the risk of clot formation, continuous heparinized saline was used to flush the side port of the introducer as well as the side port of the Micra delivery system. Intravenous heparin boluses were not administered. Single or dual antiplatelet therapies were not interrupted, however anticoagulation was discontinued for 2 days prior to the procedure, unless the patient was considered to be at high risk of thromboembolism.

### Ultrasound guided venous access

**Fig. 1:**
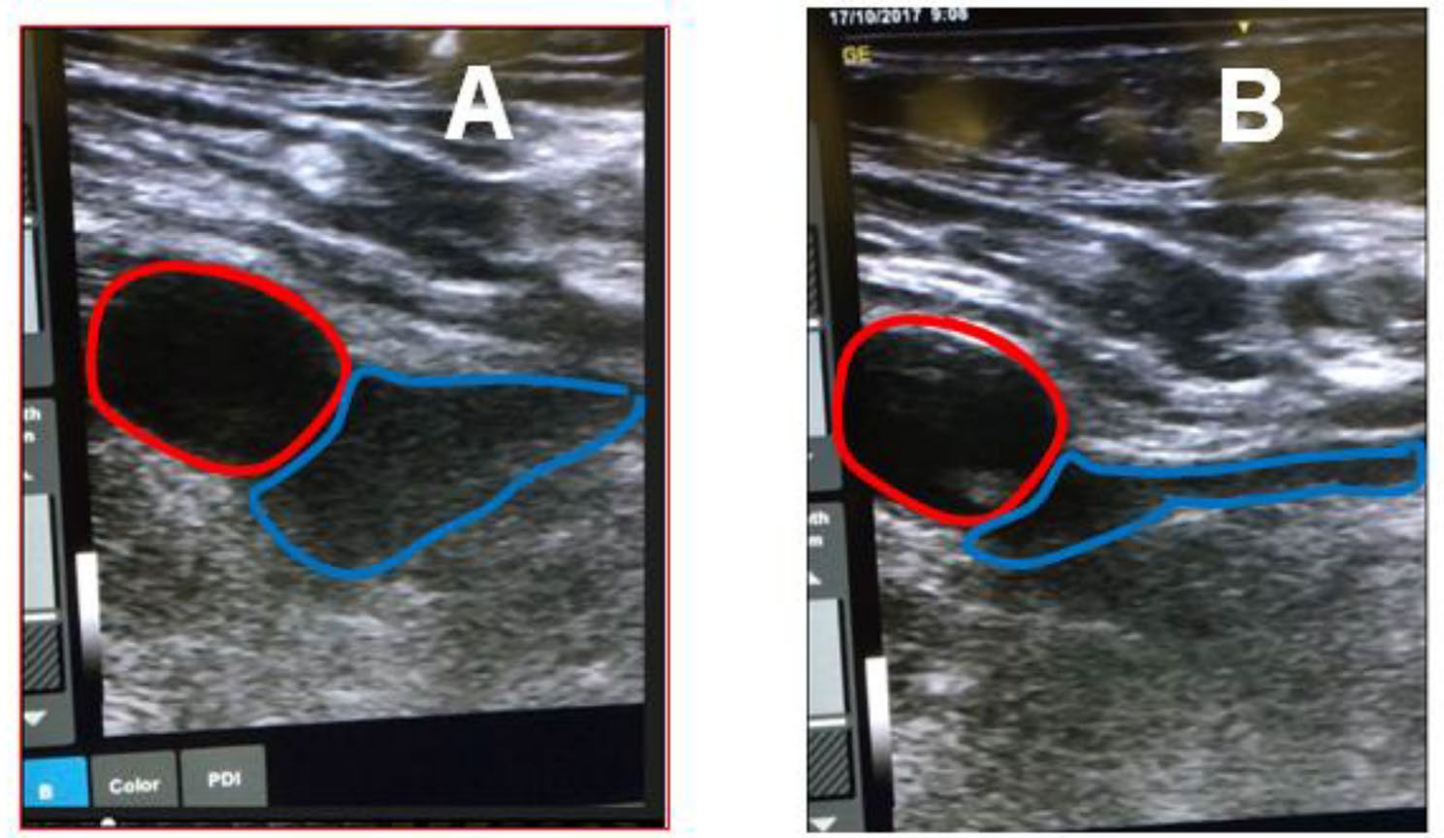
The use of ultrasound to differentiate between femoral arterial and venous circulation. **A**. Ultrasound image of right groin. Red structure represents femoral artery whereas blue structure represent femoral vein. **B**. Compression with ultrasound resulted into compressions of venous structure (blue) whereas arterial structure remained unchanged

Ultrasonography is a valuable method for evaluating the anatomy of the femoral circulation, allowing for accurate visualization of the location of the femoral vein in relation to the femoral artery and its branches. It is not uncommon for parts of the femoral vein to lie deep and posterior to the artery, as previously reported in the literature [7]. The femoral vein can be identified using either Doppler or by simple compression, in which the vein is easily compressed, as illustrated in Fig. 1.

### Z suture

**Fig. 2:**
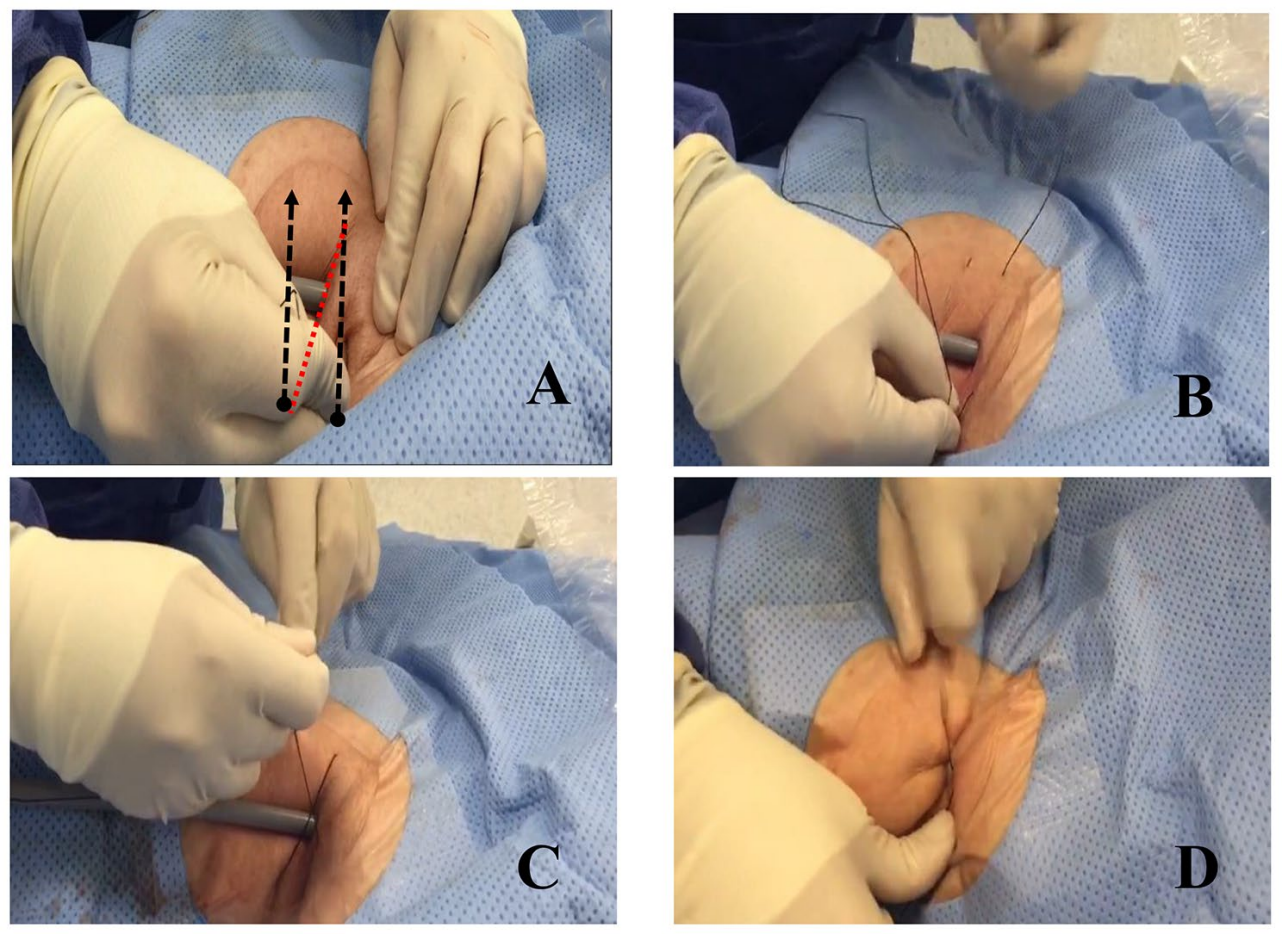
Application of Z suture for hemostasis. **A**- This shows a Micra 27Fr sheath placed in the RFV. Dotted black lines show the plane of Z suture insertion. Circle end represent entry site whereas arrow end represent exit site. Red dotted line represent suture crossing from superior exit point to inferior insertion point, which lead to formation of the Z configuration. **B**- Silk suture (Ethicon MerSilk 1, 90 mm 3/8 c cutting) attached to a curved needle is passed medially to laterally on the body, on a plane just 2 cm superior to the sheath and deep into the subcutaneous tissue. **C**- The needle is brought medially to laterally the sheath and deep into the subcutaneous tissue. This represents Z configuration prior to locking suture. Micra sheath will be removed while maintaining traction on the suture. **D**- Completed right groin Z suture after removal of Micra sheath

After the successful implantation of the Micra leadless pacemaker, a Z suture technique was employed to achieve hemostasis (Fig. 2). A silk suture (Ethicon MerSilk 1, 90 mm 3/8 c cutting) attached to a curved needle was inserted deep into the subcutaneous tissue medially to laterally, on a plane 2 cm superior to the entry site of the Micra introducer 27 Fr sheath. The needle was brought medially to laterally into subcutaneous tissue 2 cm inferior to the sheath entry site, forming a Z-shape. The Micra’s introducer sheath was then removed while maintaining traction on the suture, as depicted in Fig. 2.

## Results

### Patient characteristics

A total of 45 patients were enrolled in this study, with 53% of the cohort being male. The age range of the patients was 24 to 94 years, with an average age of 79 years (Table 1).

**Table 1:** Clinical characteristics of patients implanted with Micra Leadless Pacemaker

Patient Number	Age at Implant	Sex (M/F)	Indication	LV systolic function	Type of procedure	Type of anesthesia
**1**	88	F	AF	Normal	Elective	GA
**2**	80	M	Vascular	Normal	Non-elective	GA
**3**	39	M	Occupational	Normal	Elective	GA
**4**	72	F	AF	Mild LVSD	Elective	GA
**5**	72	M	AF	Normal	Elective	GA
**6**	69	F	Infection	Mild LVSD	Elective	GA
**7**	69	M	Infection	Normal	Elective	Local
**8**	80	F	AF	Normal	Elective	Local
**9**	51	M	Vascular	Normal	Elective	Local
**10**	80	M	Cognitive	Moderate LVSD	Non-elective	Local
**11**	70	M	AF	Normal	Elective	Local
**12**	82	F	Infection	Normal	Elective	Local
**13**	79	M	Cognitive	Normal	Elective	Local
**14**	83	F	AF	Normal	Elective	Local
**15**	91	F	AF	Normal	Non-elective	Local
**16**	91	M	AF	Normal	Non-elective	Local
**17**	85	F	AF	Normal	Elective	Local
**18**	81	F	Infection	Normal	Elective	Local
**19**	83	F	AF	Mild LVSD	Elective	Local
**20**	80	M	AF	Normal	Elective	Local
**21**	24	F	Vascular	Normal	Elective	Local
**22**	53	M	AF	Moderate LVSD	Elective	Local
**23**	77	F	AF	Normal	Elective	Local
**24**	82	M	Infection	Moderate LVSD	Elective	Local
**25**	72	M	Infection	Normal	Elective	Local
**26**	83	F	AF	Normal	Elective	Local
**27**	86	F	Cognitive	Normal	Non-elective	Local
**28**	55	M	Vascular	Normal	Elective	Local
**29**	77	F	Vascular	Normal	Elective	Local
**30**	70	M	Infection	Normal	Non-elective	Local
**31**	76	M	Infection	Normal	Non-elective	Local
**32**	94	F	Vascular	Normal	Elective	Local
**33**	79	M	AF	Normal	Elective	Local
**34**	85	F	AF	Normal	Elective	Local
**35**	87	F	AF	Normal	Elective	Local
**36**	78	F	AF	Normal	Non-elective	Local
**37**	64	M	Occupational	Normal	Elective	Local
**38**	87	M	AF	Normal	Non-elective	Local
**39**	79	M	AF	Normal	Elective	Local
**40**	91	M	AF	Normal	Elective	Local
**41**	91	F	Infection	Normal	Non-elective	Local
**42**	78	M	Vascular	Normal	Elective	Local
**43**	67	M	AF	Normal	Non-Elective	Local
**44**	86	F	AF	Normal	Elective	Local
**45**	79	M	AF	Normal	Elective	Local

M - Male, F - female, LVSD- Left Ventricle Systolic Dysfunction, GA- General anesthesia

There was a diverse range of clinical indications for implantation, with atrial fibrillation (AF), vascular, and infective causes constituting the majority of cases, at 53%, 15%, and 20%, respectively. Other indications included cognitive impairment (6%) and occupational reasons (4%).

All patients underwent an echocardiogram examination prior to the procedure. The majority of patients exhibited normal left ventricular (LV) function (86.6%), while 3 patients displayed moderate and 3 patients demonstrated mild LV dysfunction. With regard to antiplatelet therapy, a total of 34 patients were receiving either single (88%) or dual (11%) antiplatelet therapy. 75% of patients were on anticoagulation therapy, with 40% receiving Warfarin and the remaining 60% receiving a Direct Oral Anticoagulant (DOAC).

### Procedure data and complications

A total of 10 implantations were performed urgently, while the remaining were scheduled as elective cases. The majority of the cases, 39 (86.6%), received local sedation; while 6 (13.3%) cases were performed under general anesthesia.

The total procedure fluoroscopy time varied from 1 min to 26 s to 19 min and 19 s, with an average of 4 min and 8 s (Table 2).

**Table 2:** Micra Leadless Pacemaker - Procedure and Electrical characteristics

Patient Number	Implant time (mins)	Fluoroscopy (Min:Sec)	Number of Deployments	Vascular Complication	Other complication
1	69	6:39	1	No	No
2	53	5:59	2	No	No
3	55	3:54	1	No	No
4	55	7:2	1	No	No
5	66	2:3	1	No	No
6	65	4:56	1	No	No
7	25	5:41	1	No	No
8	76	2:11	1	No	No
9	69	4:2	1	No	No
10	45	6:3	1	No	No
11	74	8:19	1	No	No
12	44	3:56	1	No	No
13	55	5:12	1	No	No
14	51	3:31	1	No	No
15	105	4:11	1	No	No
16	46	4:4	1	No	No
17	25	2:39	1	No	No
18	56	6:4	2	No	No
19	50	5:45	1	No	No
20	44	2:25	1	No	No
21	66	7:41	1	No	No
22	50	11:3	1	No	No
23	44	2:5	1	No	No
24	51	6:18	1	No	No
25	59	2:5	1	No	No
26	31	14:3	1	No	No
27	57	5:47	1	No	No
28	51	1:26	1	No	No
29	60	2:4	1	No	No
30	48	3:17	1	No	No
31	105	17:35	1	No	No
32	93	19:19	1	No	No
33	17	1:42	1	No	No
34	17	2:26	1	No	No
35	26	2.08	1	No	No
36	20	2:2	1	No	No
37	35	1:97	1	No	No
38	21	1:55	1	No	No
39	90	2.42	1	No	No
40	25	2:15	1	No	No
41	19	2:08	1	No	No
42	78	4.57	1	No	No
43	79	1.41	1	No	No
44	25	2.4	1	No	No
45	20	4	1	No	No

In the cohort of 45 cases, the Micra device was successfully deployed in 43 cases on the first attempt and 2 cases required two or more deployment attempts.

Ultrasound-guided venipuncture was used for vascular access in all patients, as previously described in the methods section. Hemostasis was achieved through the use of Z suture in all cases. There were no reported immediate vascular or general complications among the patients in this study.

## Discussion

The use of conventional transvenous pacing devices has been associated with a variety of complications, including pocket and lead complications. A significant proportion of patients, approximately 10%, have reported complications such as infection, hematoma, pneumothorax, or lead dislodgement within months of the traditional implantation [4, 5]. Additionally, long-term complications associated with traditional pacing include lead fracture, venous thrombosis, and injury to the tricuspid valve [1, 4].

Recent advancements in battery chemistry and device miniaturization in electrophysiology have significantly mitigated the requirement for a subcutaneous pocket in pacing devices.

The Micra leadless pacemaker (Medtronic) is a newly developed, fully self-contained, encapsulated cardiac pacemaker that includes both battery and electrodes. It is delivered in a non-surgical manner through the use of a 27 French catheter sheath via the femoral vein to the right ventricle.

While the Micra has shown significant reduction in associated complications, a small percentage of vascular complications, reported as high as 1.2% [9], still persist. The diameter of the delivery device has been identified as the primary contributing factor to these complications.

To the best of our knowledge, no studies have been conducted to date to decrease the incidence of known vascular complications associated with Micra implantation. We conducted an observational, single-center study on a cohort of 45 patients who received Micra implantation over a 2-year period. The goal of this observational study was to assess the associated vascular complications through the use of real-time ultrasound-guided venipuncture for venous access and the Z suture technique for hemostasis.

The analysis from our study demonstrated that there were no complications associated with Micra implantation following ultrasound-guided access and Z suture for hemostasis.

It should be noted that this study is limited by its observational design in a single center, and there is no comparison group of patients who underwent Micra implantation without ultrasound-guided venous access and Z suture. Therefore, it is difficult to draw direct conclusions about the relative safety and efficacy of these techniques. Nevertheless, our data clearly showed that no vascular complications were observed in our cohort using these techniques. This suggests that the use of these techniques may lead to a significant reduction in the risk of vascular complications as compared to the 1.2% risk reported in published data [9].

### Limitations

There are several limitations inherent to this observational study. First, this is an observational study on a relatively small patient cohort. Therefore, it cannot test for any controls or draw any conclusions on superiority/ non-inferiority or establish causation with any other modalities. However, it does share a real-world view of the authors’ experience with observed complications associated with Micra implementation in a tertiary cardiac centre within a universal health care setting. Furthermore, there is no data collected on the time on US- access or achievement of hemostasis in each case. Whilst authors appreciate this as a limitation, in this observational study, all the cases were carried out by only two senior electrophysiologists (GG & KA) to reduce the risk of this potential variation. Finally, there is no information on the type of anticoagulation for our cohort of patients, which could potentially influence their post procedure bleeding risk as well as the time to achieve hemostasis. However, as this study was carried in a universal healthcare setting, the patients would have been given the same, medication-specific, instructions for pre-operative management of anticoagulation as per the UK’s National Health Service (NHS) guidelines [10].

## Conclusion

The results of our two-year experience with Micra in a single tertiary center suggest that the use of real-time ultrasound-guided venipuncture in conjunction with the Z suture technique for hemostasis may significantly decrease the known associated vascular complications associated with this procedure. However, these findings should be further supported by larger, multi-center, randomized controlled trials.

## Data Availability

data available upon request. Please contact the corresponding author (KK) to request.

## Abbreviations

DVT: Deep Vein Thrombosis
AF: Atrial Fibrillation
LV: Left Ventricle
GA: General Anesthesia
